# Systems approaches to computational modeling of the oral microbiome

**DOI:** 10.3389/fphys.2013.00172

**Published:** 2013-07-10

**Authors:** Dimiter V. Dimitrov

**Affiliations:** Diavita Ltd.Varna, Bulgaria

**Keywords:** oral microbiome, *in silico* modeling, systems biology, systems medicine, *Porphyromonas gingivalis*, biomarkers, probiotics, vaccines

## Abstract

Current microbiome research has generated tremendous amounts of data providing snapshots of molecular activity in a variety of organisms, environments, and cell types. However, turning this knowledge into whole system level of understanding on pathways and processes has proven to be a challenging task. In this review we highlight the applicability of bioinformatics and visualization techniques to large collections of data in order to better understand the information that contains related diet—oral microbiome—host mucosal transcriptome interactions. In particular, we focus on systems biology of *Porphyromonas gingivalis* in the context of high throughput computational methods tightly integrated with translational systems medicine. Those approaches have applications for both basic research, where we can direct specific laboratory experiments in model organisms and cell cultures, and human disease, where we can validate new mechanisms and biomarkers for prevention and treatment of chronic disorders.

## Systems biology of the oral microbiome

**Figure 1:**
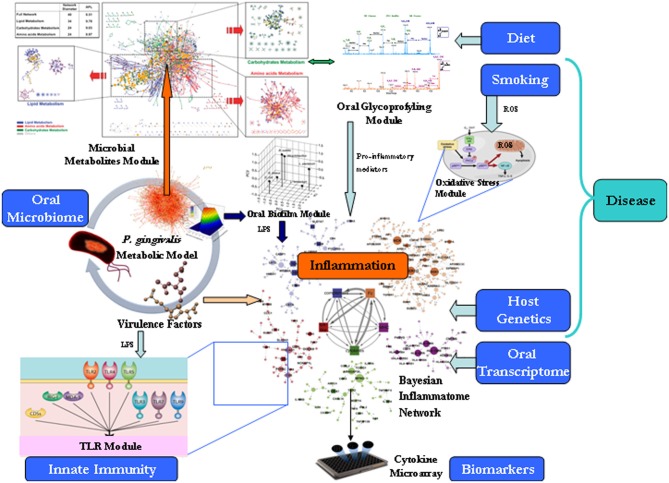
**Translational systems biology approach for modeling of the oral microbiome**. The proposed framework of different modules need be treated differently, i.e., stored differently, queried differently, and shown differently. For large data sets, it has proven efficient to follow Keim's Visual Analytics mantra: “Analyse First, Show the Important, Zoom, Filter and Analyse Further, Details on Demand” (Keim et al., 2006).

The human oral cavity is estimated to contain more than 750 bacterial species packed in biofilms (Jenkinson and Lamont, 2005; Paster et al., 2006). Three key hypotheses developed recently try to explain the connection between oral microbiota and systemic diseases. The “ecological plaque” hypothesis was formulated to link the composition and phenotypic properties of the oral microbiota associated with caries initiation and progression (Marsh, 1994). This hypothesis envisages that caries is the result of environmental changes, particularly as a result of reduced intra-oral pH as a consequence of bacterial fermentation of dietary carbohydrates. The “keystone pathogen” hypothesis was developed to explain how despite its low-level colonization of the periodontium, *Porphyromonas gingivalis* (*P. gingivalis*) causes inflammatory periodontitis through dysbiosis, i.e., an unbalancing of the relative abundance of individual components of the microbiota compared with their abundances in health (Hajishengallis et al., 2012). Finally, the “dietary carbohydrate-density” hypothesis as a link between periodontal health and metabolic health tries to explain how acellular dense carbohydrates of modern foods produce an inflammatory microbiota and from the mouth onward a low-level inflammation, with multiple elements of the metabolic syndrome (MetS) strongly correlating with circulating bacterial lipopolysaccharide (LPS) concentrations (Spreadbury, 2013). To address the questions how those emerging topics may influence human health, we propose computational systems biology modeling approaches bridging microbial genome-scale metabolic modeling, system level reconstructions of epithelial transcriptome, and molecular profiling of the dietary glycobiome (Figure 1). Biological response pathways (e.g., signaling or metabolic) often integrate with a number of other pathways, operating within a complex web of pathways. Traditional reductionism approaches that seek to explain an isolated pathway by breaking it into its component parts often cannot produce a sufficiently deep mechanistic understanding to enable predictive behaviors. This state of affairs has led to the emergence of the field of “systems biology” that seeks to develop testable models to explain the behavior of complex biological systems (Palsson, 2006). Systems biology can be defined broadly as the integration of large amounts of biological data from various sources to create one or more comprehensive models of a system to enable (1) visualization of the changes in the various working parts within a particular system (e.g., “Bayesian Network” data from changes in genes, proteins, or metabolites in response to different biological conditions), (2) visualization of the known and/or predicted interaction(s) between those parts, and (3) creation of a mathematical model of interaction paths from which testable predictions about the system can be made. This framework can also highlight areas where information is scarce; promoting the focused acquisition data that will help flesh out specific parts of the model(s). Systems approaches have been extended to model and simulate gut microbiome–host intestinal transcriptome interactions (Heinken et al., 2013). Here we describe how they may similarly shed insight on oral microbiology.

### Food molecular profiling

Dental caries is initiated by demineralization of the tooth surface due to the action of organic acid formed by dental plaque bacteria, arising from their fermentation of dietary carbohydrates. After fermentable carbohydrate intake, the plaque pH may decrease below the critical pH of 5.5, at which point human enamel undergoes demineralization, within minutes, and may remain acidified for several minutes up to several hours (Morse et al., 1989). Characteristics contributing to the ecological fitness in the oral cavity, such as utilization of different diet-derived carbohydrates, and stress tolerance to oxidative environments, should be discernible in a chewed food network model. To determine how oral microbiome functionally responds to nutrient stimuli, molecular profiling studies using food pairing approach can shed a light on individual food preferences through building a bipartite network consisting of two different types of nodes: (i) 381 ingredients used in recipes throughout the world and (ii) 1021 flavor compounds that are known to contribute to the flavor of each of these ingredients (Ahn et al., 2011). Such chemoinformatics approach is useful to determine ethnic differences in eating patterns, where North American food heavily relies on dairy products, eggs, wheat and by contrast East Asian cuisine is dominated by plant derivatives like soy sauce, sesame oil, rice, and ginger.

Targeted glycoprofyling of the oral bolus (mass of food formed in the mouth after thorough chewing) via high performance liquid chromatography-chip time-of-flight mass spectrometry can be used for monosaccharide analysis, sialic acid analysis to determine relative levels of human vs. non-human sialylation, oligosaccharide profiling, and detailed glycan structure analysis (Gamsiz et al., accepted). To specific aim will be identifying oral glycoproteins that bind to bacteria and either aid or prevent the adherence of bacteria to mucosal and tooth surfaces.

In addition, food texture of the bolus can also be measured by special equipment in research laboratories and factory quality assurance (QA) labs (Peyron et al., 2011). Texture analyzers can press, pull, pierce, squash, twist, and crush samples of food in a way which tries to mimic the end use as closely as possible. In many cases, these tests have been developed to try to mimic human senses to make the test as applicable to the product as possible, for example, to represent a biting action or a chewing action. One research group in the UK, Leatherhead Food International (LFI), is even investigating textual and structural changes in low fat foods during chewing, in other words, how the food breaks down in the mouth when it is a low-fat product. Other field of food science—food microbiology knowledge could also be extrapolated toward how ingested food changes in response to different oral microbial strains.

### The oral symbiont—network of interactions between microbial secretome and host mucosal transcriptome

Oral bacteria can adhere to salivary agglutinin, other plaque bacteria, extracellular matrix, and epithelial cell-surface receptors (Ellen et al., 1997). When explored using functional genomics approaches, the oral microbiome can permit the analysis of genes involved in colonization, survival, growth, and pathobiology of *P. gingivalis* in this unique complex environment. A common approach for building connectivity networks that integrate prior knowledge, e.g., using Kyoto Encyclopedia of Genes and Genomes (KEGG) (http://www.genome.jp/kegg/pathway/pgi/pgi00520.html) can reveal the arsenal of genes of *P. gingivalis* allowing for breakdown of sugars, and this can be further compared to carbohydrate activity gene sets of other members of the oral microbiota biofilm. Classification according to the Carbohydrate Active Enzymes (CAZy) system of Coutinho & Henrissat (Cantarel et al., 2008) will allow identification of carbohydrate-active genes including glycoside hydrolases (GH), glycosyl transferases (GT), and glycosyl esterases (CE). This will help to score the glycolysation potential of the pathogen. The genome-scale metabolic model of *P. gingivalis* demonstrates also that, upon amino acid catabolism, the organism is predicted to secrete as fermentation products succinate, propionate, and butyrate (Mazumdar et al., 2009). This would be compatible with the observation that succinate secreted by other oral pathogens is known to be used by *P. gingivalis* to produce ATP Shah and Williams (1987). Alternatively, the cellular choice of secreting mostly butyrate and propionate might be a deliberate evolved strategy for inducing harm to host cells or for supplying partner organisms with a share of useful nutrients.

Virulence factors secreted by *P. gingivalis* include gingipains and LPS. The Arg-specific cysteine proteinases (gingipains) of *P. gingivalis* exhibit complement C5 convertase-like activity, which generates high levels of C5a locally to activate the C5a receptor (Hajishengallis et al., 2012). C5aR signaling is involved in crosstalk with Toll-like receptor 2 (TLR2), which is activated in parallel by *P. gingivalis* surface ligands, and the crosstalk leads to enhanced local inflammation. This may fuel further changes to the biofilm and stabilize the transition to a disease-provoking microbiota. Metatranscriptomic analysis of oral microbial community gene expression has shown that the introduction of *P. gingivalis* into a healthy multispecies biofilm alters the pattern of community gene expression (e.g., upregulation of proteins related to growth and division, chaperones, ABC-transport systems, putative transposases, as well as numerous transcription factors).

There are numerous examples of systems models developed for various aspects of immune function activation by virulence factors, a selection of which are described below. Transcriptomic data has been most commonly used to create systems models. Examples of this approach include Ravasi et al. (2007) and Tegnér et al. (2006), who used transcriptomic data to generate a systems model of macrophage activation. Nilsson et al. (2006) used a series of mathematical and bioinformatics analyses of microarray data to study the time course of transcription factor regulation following LPS activation of macrophages. They then used bioinformatics to predict the regulation of various targets of the transcription factors, to determine transcript dynamics in the LPS network, composed of connections active during the LPS response of macrophages. This was followed by creation of a systems model demonstrating the interconnection of transcription factors and their various effectors.

Several approaches have been used to study the responses of the human oral mucosa to external stimuli, including monolayers of epithelial cells isolated from unstimulated saliva, oral squamous cell carcinomas (SCC), or primary gingival keratinocytes—organotyping of oral mucosa (Dongari-Bagtzoglou and Kashleva, 2006). For the purpose of studying the oral host–microbiome interactions, gingival keratinocytes can be used in model systems to investigate the interaction between periodontal bacteria and the epithelium in the initial stages of the periodontal disease process (2013, ]bib31a). Expression of epithelial TLRs in those cultured epithelial cells shows continuous interaction with components of oral plaque bacteria that form biofilms attached to the tooth surface. Under conditions of physiological stress (e.g., bacterial LPS and/or hypoxia), TLR signaling in epithelial cells becomes exaggerated in part through increased TLR expression. This leads to impairment in the epithelial function, increased injury, and decreased repair, resulting in mucosal inflammation.

Other environmental factors should be also taken into account when building the broad picture of interactions. In particular, smoking is major contributor to oral microbiome changes in later life and coordinate analysis in smokers reveals significantly lower taxonomic diversity, higher attachment loss and higher proportion of the anaerobic genera (Kumar et al., 2011). Mucosal cells are the first biological tissue to encounter inhaled cigarette smoke. Apparently, the oral cavity's antioxidant system fails to cope with the severe attack of reactive oxygen species originating in cigarette smoke. Application of Reverse Causal Reasoning (RCR) to cellular stress transcriptomic data has been used to build Cellular Stress Network model that describes physiological stressors and the main processes operating in response to these stressors that occur in non-diseased tissues (Schlage et al., 2011). Specifically, this network model captures the responses to oxidative, endoplasmic reticulum, hypoxic, osmotic, xenobiotic, and shear stresses. Causal relationships were constructed using a computable framework, enabling its application to the evaluation of cellular stress based on systems biology data.

### The oral inflammatome network

Bacterial components from caries activate cytokine/chemokine release from odontoblasts, dendritic cells, and/or macrophages via TLRs. Pro-inflammatory cytokines released from these cells act as autocrine and paracrine signals to amplify cytokine responses including antimicrobial peptide, cytokine, and chemokine production. The release of chemokines creates a migration gradient for immune cells to ODL while antimicrobial peptides reduce bacterial load. The minimal connectivity of genes increased in the odontoblast layer (ODL) and pulp of carious teeth network model (Horst et al., 2011) shows interactions between genes measured to be significantly upregulated and the most important candidate inflammatory signal mediators: PIK3R1, IL1R1, TLR4, ARRβ 1, CCL5, CCR5, IL8, JAK1, JAK2, RELA, and TYK2. The key receptors for inflammatory signals induced by caries in ODL appear to converge through IL1R1, CCR5, and IL8Rα/β. The gene expression data used for building this map were derived from PCR arrays and qPCR verification data of cDNA arrays. In summary, this is the first comprehensive analysis of identified potential mediators that connect local and systemic inflammation, suggesting that this type of analysis may be a useful discovery tool for novel biomarkers. Peripheral tissue trauma can initiate systemic inflammation and remote organ dysfunction. To explore how inflammation spreads the “homeostatic cytokine concentration” model was developed by Valeyev et al. (2010). In essence, the model proposes strategy for a quantitative description of multiple interactions between immune cell populations based on their cytokine production profiles. According to the dose–response curve, any given extracellular concentration of cytokine A in tissue translates to a specific extracellular concentration of cytokine B, under conditions of equilibrium. It is also possible that additional cytokine A or B production by other cell populations can also occur in tissue, resulting in cytokine A and B concentrations that do not fit the line of homeostatic equilibrium or the immune cell population considered. After such perturbation, the immune system returns to homeostasis, defined as the dose-dependent line of cytokine B production in a cytokine A-dependent manner and modulated by the cytokine removal mechanisms. Internal or external factors can change the cytokine production profiles and thereby modify the immune cell interaction parameters via feedback loops. After establishing the mechanism of chronic inflammation in the form of additional stable homeostatic level, the systems model for immune cell interactions can elucidate the causes of variety of clinical phenotypes observed in clinical practice.

## Systems medicine of the oral microbiome

“Systems medicine” is the application of systems biology approaches to medical research and medical practice (Bousquet et al., 2011). Over the past decade, a number of bioinformatics tools have been developed to predict to which parts of a microbe the immune system will react, the so-called epitopes (Rapin et al., 2006). Several methodologies are used to model immune system. Immune system models have recently been used to answer a number of immunologically relevant questions and to investigate controversial new theories or mechanisms. The computer implementation of the model (SimAthero simulator) has two main classes of parameters: the first one refers to values known from standard immunology literature; the second one collects all the parameters with unknown values which we arbitrarily set to plausible values after performing a series of tests (*tuning phase*) (Pappalardo et al., 2008). In particular, specificity is implemented in SimAthero by a bit-string polyclonal lattice method. Bit-string refers to the way the molecules and the specificity among molecules is represented, polyclonal indicates that more clones of different specificity of lymphocytes are represented and lattice means that we use a discrete lattice to represent the space, that is, the space is discrete. The set of lymphocytes receptors is represented by bit-strings of length *h* which then forms the so-called shape space. A clonal set of cells is characterized by the same clonotypic receptor, i.e., by the same bit-string of length *l*. The potential repertoire of receptors scales as 2*l*. The receptor–coreceptor binding among the entities are described in terms of matching between binary strings with fixed directional reading frame. Bit-strings represent the generic binding site between cells (through their receptors) and target molecules (through peptides and epitopes). The simulator takes care of the main interactions that happen during an immune response against atherogenesis. The model applies to the very early stage of the atherosclerosis, i.e., before a calcified plaque is formed. *In silico* experiments on two samples of one hundred virtual humans show reasonable agreements with human observations. As the model and its computer implementation is very flexible and new biological entities and interactions can be easily added to the model.

### How oral microbiome contributes to disease?

It is intriguing to study to what extend periodontopathic bacteria may directly enhance atherogenesis. Several facts provide clues of potential connection between oral bacteria and local immuno-inflammatory response. The detection frequency of *P. gingivalis*, in atherosclerostic specimens was shown to be 32% (Chen et al., 2008). Lu et al. (2008) also suggested that systemic markers of inflammation (CRP, white cell count, fibrinogen) were predictors of peripheral vascular disease and were significantly associated with periodontal attachment loss.

The increasing use of cone beam computed tomography (CBCT) in dentistry provides images in the third dimension (3D) which facilitates precise localization and prevalence of Carotid Artery Calcifications (CAC) on panoramic images in the general dental population over 50 years varies from 0.1 to 3.2% increasing with age and is substantially higher (22–37%) in populations exhibiting atherosclerotic (hypertension, cardiovascular disease, past stroke/CVA, transient ischemic attacks, or diabetes) or other (hypercholesterolemia, obesity and physical inactivity, cigarette smoking, sleep apnea, and male gender) risk factors (Lee and Kang, 2005).

**Figure 2:**
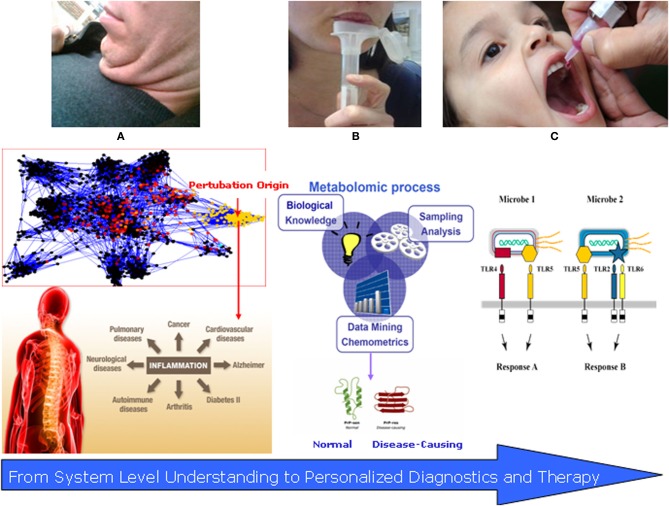
**Translational systems medicine: salivomics and vaccinomics of the oral microbiome. (A)** The “Fatty Neck”—new emerging marker of inflammatory complications; **(B)** Salivary diagnostics (salivomics)—non-invasive and applicable approach for disease detection and follow up. **(C)** Personalized vaccinomics—for prediction of protein–ligand binding regions, vaccine design using computational vaccinology of responders vs. non-responders with the overall role for disease prevention.

Recent studies have pointed to the heterogeneity of macrophages infiltrated into adipose tissue; i.e., they follow at least two different polarization states: M1 or classically activated (pro-inflammatory) macrophages, which are induced by pro-inflammatory mediators such as LPS and M2 or alternatively activated (anti-inflammatory) macrophages, which are generated *in vitro* by exposure to Th2 cytokines such as IL-4 and IL-13 (Osborn and Olefsky, 2012; Manteiga et al., 2013). Evidence has accumulated indicating that macrophages exhibit the phenotypic change from M2 to M1 polarization in obese adipose tissue, thereby accelerating adipose tissue inflammation. Local ectopic fat accumulation, as well as adipose inflammation, is now considered a significant contributor to systemic one (Thomas et al., 2012). In the context of crosstalk oral microbiome–metabolic dysregulation, the “fatty neck” could join the rest of the Metabolic Syndrome inflamed depots. Eighty percentage of the fat in the face is localized under the chin and comprises 35% of total neck fat (Figure 2A). Although considered subcutaneous fat, it is metabolically active (Cypess et al., 2013) and it is highly plausible that interacts with both oral pathogens and innate immunity. Researchers from the Framingham heart study found that even those with relatively trim waistlines appeared to be at greater risk if they had larger necks (Preis et al., 2013). In this study, average neck circumferences were 40.5 cm for men and 34.2 cm for women. As neck circumference grew, so did metabolic risk factors. For every nearly 3 cm more of neck, men had 2.2 milligrams of less good cholesterol per deciliter of blood (mg/dl) and women 2.7 mg/dl. Neck circumference was associated with cardiovascular disease (CVD) risk factors even after adjustment for visceral adipose tissue (VAT) and body mass index (BMI). These findings suggest that upper-body neck fat may be a unique, pathogenic fat depot. Recent studies have indicated a close correlation of metabolic syndrome and obesity with periodontal disease and with other chronic inflammatory diseases, including type 2 diabetes and CVD (Kuo et al., 2008; Bascones-Martínez et al., 2012; Divaris et al., 2013; Krejci and Bissada, 2013). Whether the relationship between obesity and periodontitis is causal needs to be assessed in future studies.

### Salivomics

Saliva is the oral fluid that lubricates, buffers, and protects oral tissues against decay, damage, microbial inflammation, and facilitates the remineralization of teeth. Saliva consists primarily of water, minerals, electrolytes, buffer, and proteins that are secreted by three major glands (parotid, submandibular, sublingual) and by numerous minor glands in the lip, cheek, tongue, and palate. In addition, saliva contains microbes, epithelial cells, nasal and bronchial secretions, and serum products. These components can provide clues to local and/or systemic diseases and disorders of the human body. Diagnostically, a number of findings in the past decade have prompted interest in the use of saliva as a source of biomarkers. Levels of hormones (e.g., cortisol, oxytocin) and drugs (e.g., cisplatin, nicotine, methadone) in saliva reflect their concentration in serum (Refulio et al., 2013). A significant boost to the scientific foundation and infrastructure of salivary diagnostics research came 6 years ago when the National Institute of Dental & Craniofacial Research (NIDCR) made a significant investment toward developing the use of saliva as a diagnostic tool. Saliva has since become a biofluid that is poised for translational and clinical applications. Of note is the maturation of the salivary proteome, the first implement in the diagnostic toolbox for saliva-based diagnostics. We now know there are 1166 proteins in human saliva, the functions of which range from structural binding to participation in diverse biological processes (Rathnayake et al., 2013). A second diagnostic resource in saliva has since emerged, the salivary transcriptome. Using the salivary transcriptome as a diagnostic tool, a set of 185 mRNAs was identified as “normal salivary core transcripts” (NSCT) (Fábryová and Celec, accepted). Third, the CE-TOFMS can readily and effectively be applied to salivary metabolomics. Small molecule metabolites found in human saliva can come from several sources. Depending on the method of collection, food components may be directly observed during targeted profiling of saliva. These can be solutes already dissolved in the food, like caffeine in coffee, solid components that rapidly dissolve into the saliva, like sugars from a cookie, or metabolites resulting from the action of enzymes in the saliva, like maltose from the action of α-amylase on starches (Walsh et al., 2006). For the purpose of this review, saliva metabolomics is most applicable as a direct measure of dietary input; however, systems biology integration of metabolomics data with the rest of the oral network components has not been done thus so far.

Applied salivomics can involve both insights gained from biobanking analysis, as wells as from targeted analysis in specific phenotype subjects, e.g., obese ones or smokers (Figure 2B). Data from the UK Biobank (UKB) national epidemiological study revealed that the oral salivary microbiome shows variations between subjects and suggested that the resource of 120,000 samples held in storage will be useful for phenotyping subjects and revealing potential prognostic disease biomarkers (Pramanik et al., 2012).

Human salivary circadian 27 metabolites in saliva that demonstrate a monotonic increase or decrease across the 40-h constant routine protocol (Dallmann et al., 2012). For example, the system analysis found a more than threefold increase in two fragments of the C3 complement, which is a marker of upregulation of the immune system in general.

Comparison of the salivary metabolome profile of male smokers and non-smokers revealed that citrate, lactate, pyruvate, and sucrose to be higher and formate to be lower in concentration in smokers compared with non-smokers (Takeda et al., 2009). Gender differences were also investigated, and acetate, formate, glycine, lactate, methanol, propionate, propylene glycol, pyruvate, succinate, and taurine were significantly higher in concentration in male saliva compared to female saliva (*p* < 0.05). These results show that differences between male and female, stimulated and unstimulated, as well as smoking status may be observed in the salivary metabolome.

## Personalized interventions

The modulation of pro-inflammatory cytokines in saliva may be proof of principle for interventional approaches, e.g., probiotics for combating inflammation in the oral cavity (Twetman et al., 2009). Several companies are already marketing probiotic chewing gums and would be interesting to test how they influence the level of oral pathogens in the mouth in controlled clinical settings (Vicario et al., 2011). Companies investing in clinical research with the goal of achieving an EFSA (European Food Safety Agency) approved health claim may want to re-examine their study design to ensure the data will serve its purpose. Recent consensus statement of ILSI Europe Nutrition and Immunity Task Force gives extensive overview of available inflammatory biomarkers and their utility to predict clinical outcome and highlights the need for the use of multiple markers and integrated analysis (Calder et al., 2013). In particular the authors suggest comprehensive statistical computation of cytokine pertubations over time, during and after challenge tests.

Another area of intensive research is the vaccine against *P. gingivalis* and as soon as it enters Phase I clinical trial status would be appealing to test several of the already established hypothesis and biomarkers (Jong and van der Reijden, 2010). Computational vaccinology or vaccine informatics is an interdisciplinary field that addresses scientific and clinical questions in vaccinology using computational and informatics approaches (He et al., 2013). Computational vaccinology overlaps with many other fields such as immunoinformatics, reverse vaccinology, postlicensure vaccine research, vaccinomics, literature mining, and systems vaccinology. The term “vaccinomics” or “systems vaccinology” was coined to represent a new field that integrates immunogenetics and immunogenomics with “omics-based” systems biology and immune profiling methods for the better development of next-generation of vaccines and expansion of personalized medicine studies. Literature mining can be considered as a tool within the scope of systems vaccinology. Currently, there are over 300,000 vaccine-related peer-reviewed articles cited in the PubMed literature database. This allows a comprehensive evaluation and optimization of sequence-, motif-, and SVM-based computational prediction approaches for allergens. First, the researchers collect a comprehensive dataset of known allergens and an even larger number of putative non-allergens. The prediction approaches then are integrated with this data in a web-based application that enhances allergen search and prediction.

Databases of vaccine clinical trials and vaccines in research also exist. The important vaccine components include vaccine antigens, vaccine adjuvants, vaccine vectors, and -vaccine preservatives. The vaccine antigens can be whole proteins or immune epitopes. Various *in silico* vaccine design tools are also available (He and Xiang, 2013).

The next period of medicine will focus on prevention and personalization of treatment, where identification of responders and non-responders to a given therapy will rely on early predictions based on computational modeling and stimulations. A new approach to vaccine discovery characterized as a “discover–validate–characterize–deploy” paradigm is based on the foundations of vaccinomics and personalized vaccinology and represent a range of potential components that can be assembled into a comprehensive, systems-level examination of infection/vaccination of a given pathogen (Poland et al., 2011) (Figure 2C).

In addition to combating oral inflammation with probiotics and vaccines, an intriguing study would be to estimate to what extent the anti-inflammatory effect of nicotine in the nicotine-replacement chewing gums has overall system effects, e.g., in obesity and metabolic syndrome (Lakhan and Kirchgessner, 2011).

## Conclusion

The proposed computational framework is an appropriate approach for modeling biological networks that allows *in silico* testing of new hypotheses. Specifically, the framework allows integration of new modules of interactions between components within the network. The oral microbiome systems biology–systems medicine translational model offers achievable strategy for understanding diet–microbe–host crosstalks, and can provide insights toward reducing inflammation and chronic diseases burden. The development of user-friendly and integrated bioinformatics platform for computational analysis and visualization of selected omics data in systems biology–systems medicine of the oral microbiome context is the way forward. The platform will implement the algorithms and data structures developed into one integrated tool that make these achievements directly verifiable by the community and accessible for non-expert biomedical users. Effective, reproducible, and clinically meaningful tools for combining data-driven and knowledge-based approaches to identify predictive signatures of disease are the key to future success in the biomarker and biotechnology R&D field.

### Conflict of interest statement

The authors declare that the research was conducted in the absence of any commercial or financial relationships that could be construed as a potential conflict of interest.
